# Intention to provide tobacco cessation counseling among Indonesian dental students and association with the theory of planned behavior

**DOI:** 10.1186/s12903-020-01348-4

**Published:** 2021-01-07

**Authors:** Diah Ayu Maharani, Kiarra Vashti Nadira, Febriana Setiawati, Maha El Tantawi

**Affiliations:** 1 Department of Preventive and Public Health Dentistry, Faculty of Dentistry, Universitas Indonesia, Jalan Salemba No. 4, Jakarta, 10430 Indonesia; 2 Department of Pediatric Dentistry and Dental Public Health, Faculty of Dentistry, Alexandria University, Alexandria, Egypt

**Keywords:** Dental education, Tobacco use cessation, Indonesia

## Abstract

**Background:**

Use of tobacco is a serious public health problem in Indonesia that requires a multidisciplinary approach by healthcare providers to address it. The study assessed the intentions of undergraduate students in dental schools to provide tobacco cessation counseling (TCC) and their association with the constructs of the theory of planned behavior (TPB).

**Methods:**

A cross sectional study was conducted in October 2019 using an electronic survey for dental students in Indonesian dental schools (*n* = 30). The survey assessed schools and students’ characteristics and eleven statements assessed their perspectives toward TCC based on the TPB using a 5-point Likert scale. Principal component analysis (PCA) was used to identify components within the items of perspective. Multilevel linear regression analysis was used to assess the association between intention to provide TCC and the constructs of the TPB as identified in the perspectives’ items using TPB controlling for confounders.

**Results:**

About 1288 students participated from 30 dental schools, 83.3% females with mean age = 21.5 years with average intention to provide TCC = 4.3 out of 5. They had above average positive attitude about provision of TCC being the dentist’s role (mean = 3.8 out of 5). PCA identified two components: confidence in their own abilities and perception of favorable environment with average scores = 3.2 and 2.7 out of 5. Intention to provide TCC was significantly associated with more positive attitude recognizing TCC as a dentist’s role (B = 0.10, *P* < 0.0001), greater confidence in skills to provide TCC (B = 0.17, *P* < 0.0001) and less perception of favorable environment supportive off providing TCC (B = − 0.20, *P* < 0.0001).

**Conclusions:**

Indonesian dental students’ intention to provide TCC can be explained by the constructs of the TPB. Development of dental curricula promoting professional responsibility toward TCC should be given attention. Improving students’ attitude and confidence potentially may support their patients’ efforts to quit smoking.

## Background

Smoking was the second largest risk factor for premature death and disability in the world with 20.2% of the population reported to be smoking [1, 2]. In countries with a medium to low sociodemographic index and below, there was an increase in disease burden due to smoking [3]. Indonesia has one of the highest smoking prevalence in the world. Higher prevalence of smoking is seen in people living in poverty, thereby increasing their vulnerability. On average, Indonesian smokers spend 11% of their income on tobacco which further burdens families [4]. Also, 33.8% of the population are active smokers, 65% of males are current smokers [5] and 79% of children younger than 15 years of age are affected by passive smoking [6]. The deaths caused by smoking in Indonesia account for 14.7% of total deaths, mostly from cardiovascular disease [7]. Among adolescents under 15 years of age who smoke, 8 out of 10 tried to quit but were unsuccessful due to the addictive effects of nicotine in cigarettes [8]. Smoking increases the risk of cancers [9, 10] including those of oral tissues [11, 12]. In addition, the coaggregation of *Streptococcus mutans* is higher in active smokers increasing their caries risk [13]. Smoking also results in localized inflammation of periodontal tissues [14, 15].

Health professionals are required to assess cigarette use and to provide counselling for patients to stop smoking [16]. Patients are more likely to quit smoking if health professionals encourage them to do so and help connect them with pharmacotherapy to support stopping efforts [17, 18]. However, in Indonesia, only 34.6% of smokers reported ever receiving a suggestion to stop smoking by a health professional and only 40.5% were asked about their smoking history [19]. Dentists can play a role in assessing smoking status and helping patients quit smoking [17] because of dentists’ direct and regular contact with their patients. Dentists are also the first health professional to see the effects of smoking in the oral cavity. Thus, dentists are in an ideal position to reinforce anti-smoking messages and motivate and support smokers who want to quit smoking [20]. Available evidence suggested that chairside behavioral interventions for tobacco cessation conducted by dentists may increase tobacco abstinence rates [21].

Patients’ awareness that dentist may be a smoking cessation resource, and dentists’ confidence in their knowledge and skills to providing smoking cessation services may help patients quit smoking and improve their oral health [22]. Adequate modifications in the dental education curriculum was recommended to improving dentist’s involvement in smoking cessation [23]. Education and are needed for dentists to develop their knowledge and abilities [17, 19] and foster positive attitudes toward tobacco cessation counseling (TCC) [24–26]. Most dental students are aware that TCC is within the scope of dentist services. However, lack of patients’ motivation is the most important factor hindering TCC. The education period is considered the optimum time to train students to give TCC before entering the workforce [27, 28].

The theory of planned behavior (TPB) posits that a specific behavior – such as providing TCC- is predicted by the intention to engage in this behavior. Intention, in turn, is related to the attitude toward this behavior, the perceived control over this behavior and the prevailing norms in the surrounding environment toward this behavior. The TPB was previously used to explain dentists’ intention to engage in several behaviors such as managing drug users [29], reporting suspected violence against patients [30], managing HIV- positive patients [31] and adopting preventive precautions against COVID-19 [32]. The aim of this study was to assess Indonesian dental students’ intention to provide TCC and whether this intention can be explained by the TPB. The null hypothesis was that there was no association between intention to provide TCC and the constructs of the TPB.

## Methods

In this cross-sectional study, all dental student in Indonesia (estimated number = 6132) in 30 dental schools were invited to participate in a survey in October 2019. The study protocol was approved by the Research Ethics Committee, Faculty of Dentistry, Universitas Indonesia (#66/Ethical Approval/FKGUI/VIII/2019). Written informed consent was obtained from all participants. Students were eligible to participate if they were enrolled in a Bachelor of Dentistry program in an Indonesian dental school anywhere in the country and whether the school was public or private. Students in any program level were included provided they consented to participate.

A questionnaire was developed based on previous research [28] with modifications to fit the study purpose. The questionnaire consisted of two sections. Section 1 assessed dental schools and students attributes: whether the school was in Java or other regions, whether it was public or private, student’s age in years at last birthday, gender (male or female), program level (preclinical or clinical) and smoking status (never, former and current). Section 2 included 11 statements assessing students’ perspectives regarding TCC including attitude, perceived confidence to provide TCC and support to TCC based on patients’ acceptability and dental clinic setup. These items corresponded to the constructs of the TPB: attitude toward behavior, perceived control and subjective norms [33]. Each of the 11 items was scored on a 5-point Likert scale ranging from Strongly Agree (code 1), Agree, Neutral, Disagree, Strongly Disagree (code 5).

The English questionnaire was translated to Bahasa Indonesia in accordance with the cross-cultural adaptation process guidelines [34]. Face validity of the questionnaire was assessed by 5 students to ensure clarity and wording comprehensiveness. The questionnaire was uploaded to Google Forms. A link to the electronic questionnaire was sent to the coordinators of the dental school in each of the 30 universities in Indonesia. The coordinators distributed the links to the dental students in their respective schools. The completion of the survey was unsupervised and non-obligatory. To ensure that the questionnaire was filled by students, they needed to enter their national student’s ID number. The survey period was limited to 1 month.

### Data analysis

IBM SPSS Statistics for Windows, version 23.0 (SPSS Inc., Chicago, USA) was used for data analysis. Descriptive statistics were calculated as frequencies and percentages or means and standard deviations. Principal component analysis (PCA) was used to identify the main components present within the items representing students’ perspectives regarding TCC after the calculation of Kaiser-Meyer-Olkin (KMO) measure of sampling adequacy and the *p* value of Bartlett’s test of sphericity to assess the suitability of data to PCA. Varimax rotation with Kaiser normalization was used. Loadings < 0.35 were suppressed to facilitate interpretations of loadings. Cronbach alpha was used to assess the internal consistency of the items forming the components identified by PCA. A score was calculated for the items forming each component by averaging the scores of all included items creating scores that ranged from 1 (strongly agree: least negative) to 5 (strongly disagree: most negative). Multi-level linear regression analysis was used based on the Generalized Linear Mixed models procedures in SPSS to account for the clustering of students within dental schools where students represented level 1 and schools represented level 2. The dependent variable was intention to deliver TCC on a score from 1 to 5 with scores reversed so that score 5 indicated the greatest intention. The fixed effect dependent factors were dental school attributes (region and type of school) and student’s attributes (age, gender, program level and smoking status) in addition to items as classified by PCA. Dental schools were entered as random effect variables. Regression coefficient, 95% confidence intervals (CI) and *p* values were calculated. Significance was set at 5%.

## Results

The survey was sent to 6132 and 1288 students responded (response rate = 21%). Descriptive data of the Indonesian dental students participating this study are shown in Table 1. Half the participants were from Java (50.3%). Most of them were from public dental schools (64.5%), in the clinical level of the program (52.8%) and females (83.3%) with mean age = 21.5 years. The majority never smoked (90.6%) and on average, they expressed an intention to advise patients to quit smoking (mean = 4.30 out of 5).

**Table 1:** Attributes of Indonesian dental students participating in the study

Region	Java: n (%)	648 (50.3)
	Outside Java: n (%)	640 (49.7)
Type of school	Public: n (%)	831 (64.5)
	Private: n (%)	457 (35.5)
Program level	Preclinical: n (%)	608 (47.2)
	Clinical: n (%)	680 (52.8)
Age	Mean (SD)	21.5 (1.4)
Gender	Male: n (%)	215 (16.7)
	Female: n (%)	1073 (83.3)
Student’s smoking status	Never smoked: n (%)	1167 (90.6)
	Former smoker: n (%)	103 (8.0)
	Current smoker: n (%)	18 (1.4)
Intends to advise patient to quit smoking	Mean (SD)	4.30 (0.78)

**Fig. 1:**
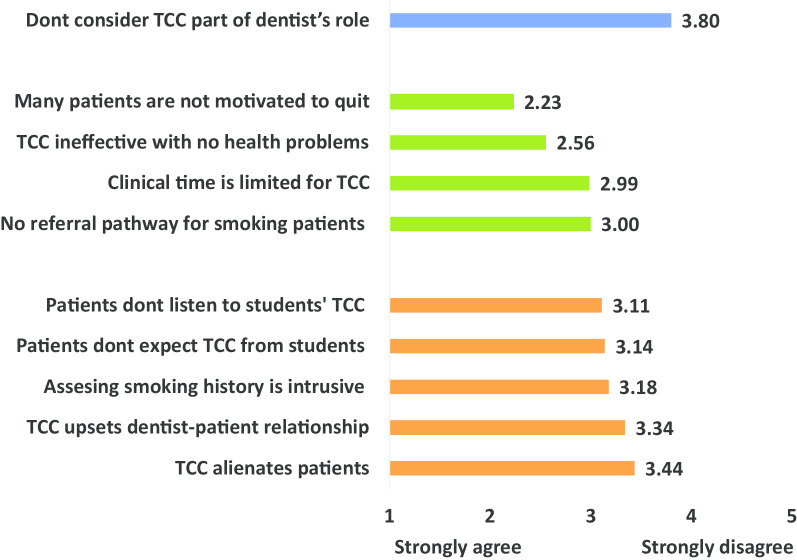
Students’ perspective regarding TCC

Figure 1 and Table 2 show that the extent of agreement/ disagreement with various statements describing students’ perspective toward TCC. The students generally disagreed (mean = 3.80) that TCC is not part of dentist’s role indicating a positive attitude toward TCC. They had a perspective that was more positive than negative regarding students’ ability to deliver TCC including that patients would listen to students delivering TCC, that they would expect TCC from students, that assessing smoking history will not be considered intrusive, will upset patient-dentist relationship or will alienate patients (mean > 3 out of 5). They had less positive perspective of patients’ interest in quitting smoking, the possibility to provide TCC because of limited clinic time or the presence of pathway to refer patients to quit (mean ≤ 3).

**Table 2:** Distribution of Indonesian dentistry students’ perspective regarding TCC

	Strongly Agree	Agree	Neutral	Disagree	Strongly Disagree
Dont consider TCC part of the dentist’s role	23 (1.8%)	83 (6.4%)	276 (21.4%)	669 (51.9%)	237 (18.4%)
Many patients are not motivation to quit	290 (22.5%)	608 (47.2%)	230 (17.9%)	123 (9.5%)	37 (2.9%)
TCC ineffective with no health problems	203 (15.8%)	495 (38.4%)	317 (24.6%)	217 (16.8%)	56 (4.3%)
Clinical time is limited for TCC	94 (7.3%)	307 (23.8%)	469 (36.4%)	355 (27.6%)	63 (4.9%)
No referral pathway for smoking patients	72 (5.6%)	312 (24.2%)	527 (40.9%)	298 (23.1%)	79 (6.1%)
Patients dont listen to dental students’ TCC	43 (3.3%)	260 (20.2%)	566 (43.9%)	349 (27.1%)	70 (5.4%)
Patients dont expect TCC from students	40 (3.1%)	256 (19.9%)	546 (42.4%)	374 (29.0%)	72 (5.6%)
Assesing smoking history is intrusive	25 (1.9%)	233 (18.1%)	580 (45.0%)	386 (30.0%)	64 (5.0%)
TCC upsets dentist-patient relationship	35 (2.7%)	243 (18.9%)	384 (29.8%)	499 (38.7%)	127 (9.9%)
TCC alienates patients	29 (2.3%)	184 (14.3%)	416 (32.3%)	514 (39.9%)	145 (11.3%)

One of the 11 items assessing perspective described the intention to give TCC and another one described attitude (considering TCC to be dentist’s role). The other nine items were included in the PCA as shown in Table 3. KMO = 0.83 and P of Bartlett’s test < 0.0001 indicating the suitability of data to PCA. The nine items loaded on two components: students’ confidence and favorable environment. Students’ confidence included 5 items with loadings ranging from 0.452 to 0.708 and favorable environment included 4 items with loadings ranging from 0.514 to 0.763. Three items loaded on both components and were included in the component where they had greater loading. The two components explained 48.8% of the variation among students’ in perspective regarding TCC.

**Table 3:** PCA of dental students’ perspectives toward TCC

Items	Student’s confidence	Favorable environment
I cannot determine a patient’s smoking history without being intrusive	0.452	
Patients do not expect TCC from a dental student	0.659	0.357
Patients do not listen to dental students during TCC	0.681	0.358
I am concerned that the message of TCC may alienate patients	0.708	
Giving unwanted TCC may upset the dentist-patient relationship	0.696	
Many tobacco-using patients do not have the motivation to quit		0.514
TCC is ineffective unless the patient has a related health problem	0.350	0.560
Clinical time is too limited to do counselling		0.756
There is no referral pathway for tobacco-using patients		0.763

Cronbach alpha for the internal consistency of the five items representing student’s confidence was 0.72 and the mean (SD) 3.2 (0.6) out of 5 indicating higher than average perception of confidence. Alpha for the internal consistency of the items representing the favorable environment was 0.65 and the mean (SD) was 2.7 (0.7) out of 5 indicating lower than average perception of favorable environment.

Table 4 shows the association between intention to provide TCC and various factors using multilevel linear regression analysis. Students in public schools expressed significantly greater intention to provide TCC (B = 01.5, 95% CI: 0.03, 0.26). Students who were never and former smokers were significantly more likely to express intention to provide TCC than those who were current smokers (B = 0.70, 95% CI: 0.34, 1.06 and B = 0.60, 95% CI: 0.22, 0.98). Students who had more positive attitude that TCC is part of dentist’s role were significantly more likely to express intention to provide TCC (B = 0.10, 95% CI: 0.05, 0.15). Students with greater confidence in their ability to provide TCC were significantly more likely to report intending to provide TCC (B = 0.17, 95% CI: 0.09, 0.25). On the other hand, students reporting greater perception of environment favorable to TCC were significantly less likely to report intention to provide TCC (B = − 0.20, 95% CI: − 0.27, − 0.13).

**Table 4:** Multilevel analysis for factors associated with intention to provide TCC

Factors	B (95% CI)	*P* value
Java versus outside Java	0.09 (− 0.02, 0.20)	0.11
Public versus private dental school	0.15 (0.03, 0.26)	0.01^a^
Clinical versus preclinical program level	−0.03 (− 0.14, 0.09)	0.62
Age	−0.02 (− 0.06, 0.02)	0.36
Female versus male	−0.02 (− 0.14, 0.10)	0.78
Never smoker versus current smoker	0.70 (0.34, 1.06)	< 0.0001^a^
Former smoker versus current smoker	0.60 (0.22, 0.98)	0.002^a^
TCC is part of dentist’s role	0.10 (0.05, 0.15)	< 0.0001^a^
Student’s confidence score	0.17 (0.09, 0.25)	< 0.0001^a^
Favorable environment score	−0.20 (−0.27, − 0.13)	< 0.0001^a^

B: regression coefficient, *CI* Confidence interval, ^a^ statistically significant at *P* < 0.05, Schools included as random effect variable

## Discussion

Dental Indonesian students’ intention to deliver TCC was associated with more positive attitude towards dentist’s role in TCC, greater confidence in the ability to deliver TCC and less favorable supporting environment concerning patients’ motivation and clinic setup. The null hypothesis of the study is, thus, rejected and the intention to deliver TCC can be explained by the constructs of the TPB.

In the present study, dental students’ intention to deliver TCC was high. This high level agrees with another study in Hong Kong [28] reporting that 96% of dental students indicated that they would advise patients to stop smoking later in their careers. It was also found that 72% agreed that counseling to motivate patients to stop smoking is part of the dentist’s role [28]. A systematic review [27] reported that 54 to 96% of students in several studies planned to provide TCC. In the present study, the attitude regarding dentists’ role in TCC was positive. Furthermore, other studies reported that 40 to 98.1% of dental students agreed that dental professionals had a role in TCC [27].

Student’s confidence was positively associated with intention to provide TCC in the present study. Adequate training increases students’ confidence in their ability to help patients quit smoking and provides a structured approach that they can follow to achieve this objective. Several techniques [35] are available to enable dental professionals’ to help their patients quit smoking including behavioral interventions such as the 5As intervention for patients who are ready to quit: Ask, Advise, Assess, Assist, Arrange and the 5Rs for patients who are not yet ready to quit (Relevance, Risks, Rewards, Roadblocks and Repetitions) [36]; nicotine replacement therapy (NRT), pharmacological therapy and referral to specialized centers. These methods need to be incorporated into dental curricula and continuing education activities. The study findings agree with Ching et al. [28] who reported that what most inhibited dental students from counseling patients to quit smoking was the feeling of not having sufficient skills to provide TCC.

In the present study, the intention to provide TCC was negatively associated with the presence of a favorable environment that was more strongly loaded by clinic setup factors than patient motivation factors. This may be explained by dental students’ perceiving no need for their own efforts to help patients quit smoking due to the presence of a clinic setup that helps achieve this without their direct involvement. On the other hand, Pendharkar et al. [37] reported that lack of time in the clinic prevented dental students from providing interventions for patients to quit smoking. The inverse association between patient motivation and dental students’ intention to provide TCC in the present study disagrees with Ching et al. [28] and a systematic review [27] reporting that patients’ lack of motivation was a barrier against their involvement in TCC. Motivating patients to quit smoking is a skill that can be developed in dental students using proper techniques such as motivational interviewing [38, 39]. It is important that dental students learn the importance and acquire the skills for such proactive, upstream approaches to protect against the harmful oral health effects of tobacco rather than follow a reactive, downstream approach by treating tobacco effects on the hard and soft tissues of the oral cavity. In addition, these skills help integrate dental students and dentists in the healthcare team to fight the deleterious effects of tobacco on health and wellbeing.

The present study showed that dental students’ intention to provide TCC was associated with the constructs of the TPB. Similarly, the TPB was previously used to explain compliance with tobacco free policy among university students [40] and predict smoking behaviors among students [41]. The advantages of using TPB for this study is that TPB provides a conceptual framework to explain behavior, which is crucial in guiding the development of training to prepare dental students to meet the needs of TCC [29]. Furthermore, TPB is characterized by several features that may help explain its widespread use as a model for the prediction and change of behavior [42]. TPB focuses directly on the determinants of behaviors, offers a clearly specified structural model, which provides a conceptual framework and theoretical constructs for analyzing determinants of the behavior under consideration [42]. The present study thus fills a knowledge gap by providing insights into the association between the TPB and intention to offer TCC among dental students in Indonesia.

In this study, students who did not smoke or were former smokers had greater intention to deliver TCC than students who were current smokers. Previous studies also stated that dentist smoking was a significant factor associated with giving TCC to patients [43–45]. Non-smoking dentists believe that dentists should help patients stop smoking, set good examples, have an influence on policy, effectively facilitate and provide treatment for smoking cessation. Conversely, dentists who smoke were reported to not record a patient’s smoking history or provide counseling to stop smoking in patients [43, 44]. Therefore, interventions are needed to help dentists who smoke quit smoking. Further results showed that students in public university expressed significantly greater intention to provide TCC. Admission into public universities is highly competitive, therefor public university students might have positive attitude, awareness [46] and literacy [47].

The large number of dental schools as well the inclusion of dental students from various dental schools are major strengths of the study. There are some limitations, however. The cross-sectional design suggests association but cannot support causality. Future longitudinal studies are needed to follow reported intention to deliver TCC over time and ascertain whether it would be associated with delivery of TCC. Some dental schools had lower response rate than others which may be attributed to the lack of onsite coordinators or their inability to collect adequate responses. The overall response rate was also low and may have introduced some selection bias affecting the representativeness of the study for Indonesian dental students. However, this low rate was previously reported for electronic surveys [48]. In addition, volunteer bias cannot be ruled out. It may suggest possible overestimation of intention to deliver TCC and underestimation of the percentage of students who were current smokers. For example, the percentage of current smokers in the present study was much lower than that of the general adult population in Indonesia recorded at 34.8% [7] which may be partly attributed to the greater awareness of dental students than the general population and partly to under reporting.

According to the clinical practice guideline for treating tobacco use and dependence, clinicians are responsible for providing repeated interventions, documenting tobacco use status and providing brief cessation counselling [16]. It was reported that 70% of smokers see a physician, and almost one third see a dentist [16]. Therefor it is important for dental students to learn and implement evidence-based guidelines, which provides recommendations for clinical approaches in different settings. Training of dental students on tobacco cessation in dental schools is indispensable. Academic institutions play a vital role in tobacco control strategies through tobacco dependence intervention education. Dental schools are recommended to incorporate tobacco prevention and cessation intervention education into their curricula. Students can implement brief tobacco cessation counseling and can affect healthier patient outcomes [39]. As future licensed professionals, graduates will have knowledge, abilities and confidence to offer their patients brief TCC tailored to their specific health concerns and address an identified global health priority.

## Conclusions

Indonesian dental students’ intention to provide TCC can be explained by the constructs of the TPB. The findings have implications for dental students’ training which should aim at fostering positive attitude and developing confidence in their abilities to provide TCC. In addition to the traditional focus on increasing students’ knowledge, educational programs should also aim to promote soft learning outcomes. Furthermore, dental students who are current smokers seem to have minimal intention of helping others quit smoking. Thus, TCC for dental students themselves seems to be a priority before these students can help their patients.

## Data Availability

The raw data are available from the authors to any author who wishes to collaborate with us.

## Abbreviations

TCC: Tobacco Cessation Counseling
TPB: Theory of Planned Behavior
PCA: Principal component analysis
